# A Variant in COX-2 Gene Is Associated with Left Main Coronary Artery Disease and Clinical Outcomes of Coronary Artery Bypass Grafting

**DOI:** 10.1155/2017/2924731

**Published:** 2017-01-18

**Authors:** Hanning Liu, Zhengxi Xu, Cheng Sun, Dachuan Gu, Xiao Teng, Yan Zhao, Zhe Zheng

**Affiliations:** State Key Laboratory of Cardiovascular Disease, Fuwai Hospital, National Center for Cardiovascular Diseases, Chinese Academy of Medical Sciences and Peking Union Medical College, Beijing, China

## Abstract

As a particular severe phenotype of coronary artery disease (CAD), left main coronary artery disease (LMCAD) is heritable. Genetic variants related to prostaglandin metabolism are associated with LMCAD. Cyclooxygenase-2 (COX-2), a key synthase in prostaglandin pathways, displays high density in atherosclerotic lesions and promotes early atherosclerosis in CAD progression. We hypothesized that genetic variants in COX-2 gene contribute to LMCAD phenotype susceptibility compared to more peripheral coronary artery disease (MPCAD). In this study, we genotyped COX-2 rs5275, rs5277, and rs689466 of 1544 CAD patients undergoing coronary artery bypass grafting (CABG) and found that rs5277 C allele carriage was associated with LMCAD (adjusted OR: 1.590; 95% CI: 1.103~2.291; *p* = 0.013). Furtherly, long-term follow-up data suggested that rs5277 C allele carriage increased risk of major adverse cardiac and cerebrovascular events (MACCE) in the whole cohort (adjusted HR: 1.561; 95% CI: 1.025~2.377; *p* = 0.038) and LMCAD subgroup (adjusted HR: 2.014; 95% CI: 1.036~3.913; *p* = 0.039) but not in MPCAD subgroup (adjusted HR: 1.375; 95% CI: 0.791~2.392; *p* = 0.259). In conclusion, we demonstrate that COX-2 rs5277 C allele increases the risk of left main coronary artery lesion and is also correlated with poor prognosis of LMCAD patients with CABG therapy.

## 1. Introduction

Left main coronary artery (LMCA) arises from the left aortic sinus and bifurcated into left anterior descending (LAD) and left circumflex (LCx) arteries, which provides approximately 75% blood supply for left ventricular (LV) cardiac mass in right dominant or balanced patients and 100% in patients with left dominant type. As a result, lesions in LMCA will reduce main flow to LV, place patients at high risk of LV dysfunction, and increase occurrence of life-threatening events [1, 2].

Recently, studies revealed that angiographically diagnosed left main coronary artery disease (LMCAD) was heritable [3, 4]. Among patients with coronary artery disease (CAD), the most hazardous localization, especially LMCA, displays a high heritability [3]. Further study confirmed that asymptomatic siblings of patients with LMCAD have increased risks for future cardiovascular events than healthy siblings of patients with other manifestations of CAD [4]. Moreover, many studies have identified that several genetic variants were associated with LMCA lesions [5–9].

Cyclooxygenase-2 (COX-2) is an isoform of cyclooxygenase, which synthesizes arachidonic acid (AA) into hydroperoxy endoperoxide PGG_2_ and its subsequent reduction to the hydroxy endoperoxide PGH_2_, the precursor for eicosanoid synthesis [10]. The eicosanoid is known to be involved in the pathogenesis of inflammatory disorders, which play key roles in atherosclerosis process [11]. Furtherly, expression of COX-2 is augmented in atherosclerotic lesions but not in normal arteries [12]. Within atherosclerotic lesions, COX-2 is dominantly expressed in macrophage and foam cells, which suggests an important participation of COX-2 in the process of atherosclerosis [13]. Previous studies also demonstrated that COX-2 promotes early atherosclerotic lesion formation in mouse models [14, 15].

On the basis of biological and pathological significance of COX-2, we hypothesize that genetic variations in COX-2 gene contribute to the heritability of LMCAD. To test this hypothesis, we detected 3 genetic variations (COX-2 rs5275, rs5277, and rs689466) in a hospital-based case-only cohort and analyzed the correlation of COX-2 genetic polymorphisms and LMCAD.

## 2. Materials and Methods

### 2.1. Study Subjects

The study protocol received approval from the Review Board of Peking Union Medical College (Beijing, China), and we have strictly complied with the World Medical Association Declaration of Helsinki. All patients provided written informed consent to be involved in the study.

This study involved 1544 patients with coronary heart diseases. Patients were recruited from the Cardiovascular Institute and Fuwai Hospital, Chinese Academy of Medical Sciences, and Peking Union Medical College (Beijing, China) between December 2007 and December 2011. All patients were genetically unrelated ethnic Han Chinese and were diagnosed using angiography and confirmed by surgery. Well-trained clinical research staffs collected the data and subsequently double-entered data into computer databases. Baseline information on personal and clinical characteristics, as well as in-hospital events after CABG, was complete for all 1544 patients involved in the study. LMCAD was defined as a lesion compromising the lumen by >50%, proximal to the bifurcation, including ostial stenosis. Lesions compromising the lumen by >50% outside of the LMCA were defined as more peripheral coronary artery disease (MPCAD). All participants in the present study were followed up by visit or telephone by the research staff using standard procedures and forms as previously described [16, 17]. The clinical endpoint of this study was a composite of major adverse cardiac and cerebrovascular events (MACCE, i.e., death from any cause, stroke, myocardial infarction, or repeat revascularization) [16].

### 2.2. DNA Isolation and Genotyping

Blood samples were collected by experienced nurses using vacuum tubes containing ethylenediamine tetra-acetic acid (EDTA). Then, we isolated genomic DNA from whole blood using the Wizard Genomic DNA Purification Kit (Promega, Madison, WI). Quality control of sample DNAs was conducted by performing polymerase chain reaction (PCR) and analysis on a 3% agarose gel and visualized by ethidium bromide staining. Genotyping of the single nucleotide polymorphisms (SNPs, COX-2 rs5275, rs5277, and rs689466) was performed by MALDI-TOF MS support from CapitalBio Corporation (Beijing, China) [18–20]. Sample transfer was completed by MassARRAY Nanodispenser (Sequenom) to a 384-well spectroCHIP (Sequenom) and then analyzed by MALDI-TOF-MS. MassARRAY RT genotype-calling software (version 3.1; Sequenom) was used to call each genotype in real time eliminating a time-consuming process.

### 2.3. Statistical Analyses

Differences in demographics, variables, and genotypes of the 3 polymorphism variants were evaluated using a chi-squared test for discrete variable or Student's *t*-test for continuous variable. The associations between the 3 SNPs and risk of CAD phenotype were estimated by computing odds ratios (ORs) and 95% confidence intervals (CIs) using univariate or multivariate logistic regression analyses and by using ORs. Univariate or multivariate Cox proportional hazards regression models were preformed to estimate the crude hazard ratios (HRs) or adjusted HRs and their 95% CIs. Age, sex, BMI, smoking status, hypertension, hyperlipidemia, DM, peripheral arterial disease, and EF were used in the adjusting models. All statistical analyses were done with SPSS software (version 19.0; SPSS Inc., Chicago, IL, USA).

## 3. Results

### 3.1. Patients Characteristics

In this cohort, 488 patients (31.6%) were diagnosed with LMCAD, and 1057 patients (68.4%) had MPCAD. Patients with LMCAD were older than those with MPCAD (62.28 versus 60.88, *p* = 0.003). Male patients were more common in LMCAD patients compared to those in MPCAD group (83.3% versus 78.6%, *p* = 0.033). For other baseline variables, for instance, body mass index (BMI), smoking status, hypertension, hyperlipidemia, diabetes mellitus, peripheral arterial disease, and ejection fraction, there were no significant differences between LMCAD and MPCAD groups. During follow-up, we recorded the medication of patient and found that, between two groups, the usage of aspirin, ACEI, *β*-blocker, diuretics, calcium channel blocker, statins, clopidogrel, and nitroglycerin showed no significant differences (Table 1).

### 3.2. Association between Single Nucleotide Polymorphisms (SNPs) and LMCAD

We successfully genotyped all the three SNPs with genotyping values at least more than 97%. All these three SNPs were classified into two genotypic groups, distinguished by the absence or presence of at least one copy of the minor allele (homozygote major versus homozygote minor and heterozygote), and statistical analyses were performed based on these two groups. Only rs5277 exhibited significant correlation with LMCAD. Among LMCAD patients, 11.3% carried rs5277 C allele, while 7.5% patients with MPCAD carried rs5277 C allele. CAD patients carrying rs5277 C allele had 1.575 times risk of LMCAD than those who did not carry rs5277 C allele (95% CI: 1.096~2.262; *p* = 0.014). When adjusted by age, sex, BMI, smoking, hypertension, hyperlipidemia, DM, peripheral arterial disease, and ejection fraction, rs5277 C allele carriage also manifested association with LMCAD (adjusted OR: 1.590; 95% CI: 1.103~2.291; *p* = 0.013). For rs5275 and rs689466, there was no significant correlation with LMCAD (Table 2).

### 3.3. COX-2 rs5277 C Allele Carriage and the Prognosis of Patients Undergoing CABG

**Figure 1:**
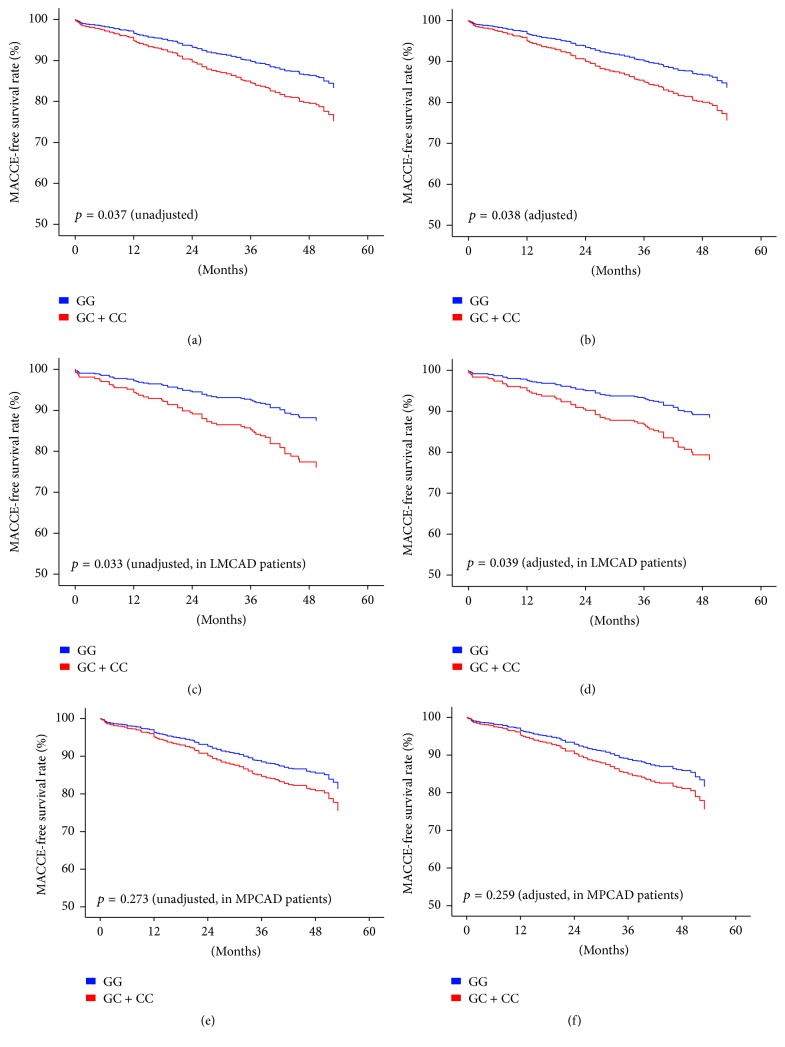
Kaplan-Meier curves of MACCE-free survival rate according to COX-2 rs5277 genotypes. Survival analyses of MACCE-free survival rate according to COX-2 rs5277 genotype in (a & b) the whole cohort ((a) unadjusted, *p* = 0.037; (b) adjusted, *p* = 0.038), (c & d), LMCAD subgroup ((c) unadjusted, *p* = 0.033; (d) adjusted, *p* = 0.039), and (e & f) MPCAD subgroup ((e) unadjusted, *p* = 0.273; (f) adjusted, *p* = 0.259). MACCE, major adverse cardiac and cerebrovascular events; CABG, coronary artery bypass grafting; LMCAD, left main coronary artery disease; MPCAD, more peripheral coronary artery disease.

To further study whether rs5277 affects clinical outcomes in CAD patients, we performed survival analyses in all the CAD patients, LMCAD subgroup, and MPCAD subgroup. All the patients in this cohort were followed up for a median period of 43.8 (40~48.7) months. In all CAD patients, rs5277 C allele carriage increased hazard of long-term MACCE after CABG (HR: 1.561; 95% CI: 1.027~2.375; *p* = 0.037). As rs5277 C allele increased the risk of LMCAD, we studied the association of rs5277 and prognosis in LMCAD subgroup and MPCAD subgroup, respectively. Interestingly, we found out that rs5277 C allele carriage only affected long-term prognosis in LMCAD patients (HR: 2.042; 95% CI: 1.057~3.942; *p* = 0.033) but not in MPCAD patients (HR: 1.362; 95% CI: 0.784~2.364; *p* = 0.273). In addition, we performed multivariate Cox proportional hazards regression in all CAD patients, LMCAD subgroup, and MPCAD subgroup. After adjusting, the correlations of rs5277 and prognosis in all CAD patients (adjusted HR: 1.561; 95% CI: 1.025~2.377; *p* = 0.038) or LMCAD patients (adjusted HR: 2.014; 95% CI: 1.036~3.913; *p* = 0.039) were still significant, and, in MPCAD patients, rs5277 C allele did not associate with adverse events (adjusted HR: 1.375; 95% CI: 0.791~2.392; *p* = 0.259) (Table 3 and Figure 1).

## 4. Discussion

In this study, we evaluated three polymorphisms in COX-2 gene and the prevalence and prognosis of left main coronary artery lesion in CAD patients. With a moderate size cohort of 1544 CAD patients, we found that COX-2 rs5277 C allele carriage is associated with higher prevalence of LMCAD, and this risk allele also increases long-term risk of adverse events in both CAD and LMCAD patients. To the best of our knowledge, this study is the first one to demonstrate the relationship of genetic polymorphisms and prevalence and prognosis of LMCAD.

Our findings are supported by previous reports, which demonstrated that LMCAD has a higher heritability and identified several genetic variants associating with increase of LMCAD risk [3–9]. Studies in monozygotic twins also revealed that 75% of twin pairs display concordance for LMCAD, whereas only 25% are concordant for MPCAD [21, 22]. Direct evidences of the correlation between genetic variations and LMCAD were also provided. Wang and colleagues reported that polymorphisms in an intron of a tumor suppressor gene, LSAMP, are associated with risk of LMCAD [5]. Notably, Bousoula et al. demonstrated that CYP8A1 polymorphism is associated with higher risk of LMCAD [7]. CYP8A1 gene encodes prostaglandin I_2_ (PGI_2_) synthase, which is a key enzyme in the pathway of prostaglandin metabolism. The substrate of PGI_2_ synthase, PGH_2_, is the product of COX-2 [10]. Together with this evidence, our finding strongly indicates that prostaglandin metabolism affects left main coronary artery lesion. PGI_2_ is a vasodilator inhibiting platelet aggregation, leukocyte adhesion, and vascular smooth muscle cell proliferation; thus, PGI_2_ plays important role in regulating the homeostasis of cardiovascular system and in preventing atherosclerosis [23].

Capodanno et al. identified that left main coronary lesions are more likely to present with atherosclerotic disease and significant stenosis [24]. Consistently, the expression of COX-2, a key synthase in the pathway of prostaglandin metabolism, is augmented in atherosclerotic area [12] and more specifically in macrophages and foam cells [13]. These findings indicated that COX-2 plays an important role in the progression of left main coronary lesion. As an isoform of cyclooxygenase, COX-2 is mainly activated under inflammatory stimuli [10, 25]. Since atherosclerosis is an inflammatory disease [11], it is reasonable that, in the long-term endurance of left main coronary lesion, COX-2 plays an important role in prognosis among LMCAD patients. Moreover, our findings that COX-2 rs5277 C allele increases risk of adverse events only in LMCAD patients but not in MPCAD patients are consistent with the correlation of COX-2 rs5277 C allele carriage and increased prevalence of LMCAD. The data mutually strengthened each other. In addition, our study enrolled patients undergoing CABG, which has relatively higher prevalence of LMCAD than normal CAD patients. By analyzing CABG patients, it is more likely to find the underlying genetic basis of LMCAD.

Several limitations in this study should be addressed. First, as a hospital-based study, the selection bias was unavoidable. Further work based on community or population beyond hospital ought to be carried out to elucidate this issue much clearly.

Second, the correlation between COX-2 and LMCAD was based on just three SNPs, which limited the comprehensive view of the genetic variability underlying these phenotypes; further, fine-mapping analyses might give insights into the pathophysiological mechanisms underlying LMCAD. Finally, replication of these results in different population is necessary to confirm our findings.

In conclusion, this study proves that genetic variant COX-2 rs5277 C allele increases the risk of left main coronary artery lesion and also correlated with poor prognosis of LMCAD patients with CABG therapy. These findings raise the potential possibility that patients carrying COX-2 rs5277 C allele should be identified and give special treatments to prevent the progression of left main coronary artery lesion and the occurrence of adverse events.

## Figures and Tables

**Table 1:** Baseline characteristics of patients.

Variable	LMCAD (*n* = 488)	MPCAD (*n* = 1056)	*p* value
Age, years	62.28 (±8.47)	60.88 (±8.68)	**0.003 **
Male, *n* (%)	407 (83.4)	830 (78.6)	**0.028 **
Body mass index, kg/m^2^	25.51 (±3.15)	25.87 (±5.51)	0.183
Smoking status, *n* (%)	248 (50.8)	536 (50.8)	0.982
Hypertension, *n* (%)	320 (65.6)	706 (66.9)	0.620
Hyperlipidemia, *n* (%)	329 (67.4)	713 (67.5)	0.969
Diabetes mellitus, *n* (%)	154 (31.6)	360 (34.1)	0.326
Peripheral arterial disease, *n* (%)	15 (3.1)	19 (1.8)	0.113
Ejection fraction, %	60.20 (±8.07)	59.38 (±8.87)	0.071
Medications, *n* (%)			
Aspirin	464 (95.1)	992 (93.9)	0.368
ACEI	137 (28.1)	310 (29.4)	0.605
*β*-Blocker	281 (57.6)	623 (59.0)	0.600
Diuretics	30 (6.1)	49 (4.6)	0.211
Calcium channel blocker	140 (28.7)	342 (32.4)	0.145
Statins	339 (69.5)	697 (66.0)	0.178
Clopidogrel	31 (6.4)	68 (6.4)	0.948
Nitroglycerin	280 (57.4)	595 (56.3)	0.703

Values are mean ± SD or *n* (%). ACEI indicates angiotensin-converting enzyme inhibitors; LMCAD, left main coronary artery disease; MPCAD, more peripheral coronary artery disease.

**Table 2:** Main effects of COX-2 SNPs on LMCAD risk.

SNP	Genotyping value	Genotype	*N*		Unadjusted		Adjusted	
			LMCAD (%)	MPCAD (%)	OR (95% CI)	*p* value	OR (95% CI)	*p* value
rs5275	97.4%	TT	307 (64.6)	665 (64.6)	1.000 (0.796~1.255)	0.998	0.999 (0.795~1.256)	0.994
		TC + CC	168 (35.4)	364 (35.4)				
rs5277	99.9%	GG	432 (69.3)	977 (92.5)	1.575 (1.096~2.262)	**0.014**	1.590 (1.103~2.291)	**0.013**
		GC + CC	55 (11.3)	79 (7.5)				
rs689466	98.8%	AA	140 (28.9)	309 (29.7)	1.079 (0.827~1.406)	0.577	1.086 (0.831~1.420)	0.544
		AG + GG	345 (71.1)	731 (70.3)				

Values are *n* (%). CI, confidential interval; COX-2, cyclooxygenase-2; LMCAD, left main coronary artery disease; MPCAD, more peripheral coronary artery disease; OR, odds ratio; SNP, single nucleotide polymorphism.

**Table 3:** Cox regression analyses of rs5277 and the MACCE after CABG in all patients and LMCAD and MPCAD subgroups.

Subgroup	*N*		Unadjusted			Adjusted		
	With MACCE	Without MACCE	HR	95% CI	*p* value	HR	95% CI	*p* value
All patients	199	1344	1.561	1.027~2.375	**0.037**	1.561	1.025~2.377	**0.038**
LMCAD	57	430	2.042	1.057~3.942	**0.033**	2.014	1.036~3.913	**0.039**
MPCAD	142	914	1.362	0.784~2.364	0.273	1.375	0.791~2.392	0.259

CABG, coronary artery bypass grafting; CI, confidential interval; HR, hazard ratio; LMCAD, left main coronary artery disease; MACCE, major adverse cardiac and cerebrovascular events; MPCAD, more peripheral coronary artery disease.
